# Structure of mouse coronavirus spike protein complexed with receptor reveals mechanism for viral entry

**DOI:** 10.1371/journal.ppat.1008392

**Published:** 2020-03-09

**Authors:** Jian Shang, Yushun Wan, Chang Liu, Boyd Yount, Kendra Gully, Yang Yang, Ashley Auerbach, Guiqing Peng, Ralph Baric, Fang Li

**Affiliations:** 1 Department of Veterinary and Biomedical Sciences, University of Minnesota, Saint Paul, Minnesota, United States of America; 2 Department of Epidemiology, University of North Carolina, Chapel Hill, North Carolina, United States of America; 3 College of Life Science and Technology, Huazhong Agricultural University, Wuhan, China; The University of Hong Kong, HONG KONG

## Abstract

Coronaviruses recognize a variety of receptors using different domains of their envelope-anchored spike protein. How these diverse receptor recognition patterns affect viral entry is unknown. Mouse hepatitis coronavirus (MHV) is the only known coronavirus that uses the N-terminal domain (NTD) of its spike to recognize a protein receptor, CEACAM1a. Here we determined the cryo-EM structure of MHV spike complexed with mouse CEACAM1a. The trimeric spike contains three receptor-binding S1 heads sitting on top of a trimeric membrane-fusion S2 stalk. Three receptor molecules bind to the sides of the spike trimer, where three NTDs are located. Receptor binding induces structural changes in the spike, weakening the interactions between S1 and S2. Using protease sensitivity and negative-stain EM analyses, we further showed that after protease treatment of the spike, receptor binding facilitated the dissociation of S1 from S2, allowing S2 to transition from pre-fusion to post-fusion conformation. Together these results reveal a new role of receptor binding in MHV entry: in addition to its well-characterized role in viral attachment to host cells, receptor binding also induces the conformational change of the spike and hence the fusion of viral and host membranes. Our study provides new mechanistic insight into coronavirus entry and highlights the diverse entry mechanisms used by different viruses.

## Author summary

Coronaviruses recognize many receptors using their envelope-anchored spike protein. The role of receptor binding in coronavirus entry into host cells is a fundamental question in virology. Mouse hepatitis coronavirus (MHV) is unique among all coronaviruses in that it uses the N-terminal domain (NTD) of its spike protein to bind a protein receptor CEACAM1a. While extensive research has been performed on the cell entry mechanisms of coronaviruses that use a different domain of their spike protein for receptor binding, the cell entry mechanism for MHV is still elusive. Here we determined the cryo-EM structure of MHV spike protein complexed with CEACAM1a. The structure reveals unique features of receptor binding by MHV spike that facilitate the structural changes of MHV spike and promote cell entry of MHV. We further confirmed the structural results with biochemical and negative-stain EM analyses. These results suggest that receptor binding plays dual roles in MHV entry: it promotes both viral attachment to host cells and the fusion of host and viral membranes. Our study provides insight into the molecular mechanism of MHV entry, demonstrating how cell entry of MHV has been adapted to its unique way of receptor binding.

## Introduction

A distinctive feature of coronaviruses is that they have evolved to recognize a variety of receptors including both protein receptors and sugar receptors [1]. Coronaviruses enter cells through a two-step process: they first recognize a host-cell-surface receptor for viral attachment and then fuse viral and host membranes for entry. Receptors not only determine the viral attachment step, but also play important roles in the membrane fusion process [2]. How the diverse receptor recognition patterns of coronaviruses affect their cell entry process at the molecular level presents a fundamental and critical question in virology. Mouse hepatitis coronavirus (MHV) differs from all other known coronaviruses in its mechanism of receptor recognition. This study investigates the unique roles of receptor recognition in MHV entry.

Coronaviruses are large, enveloped and positive-stranded RNA viruses that infect many mammalian and avian species and cause respiratory, enteric, gastrointestinal, and neurological diseases [3, 4]. They can be divided into four genera: α, β, γ, and δ [5]. For coronaviruses from all four genera, an envelope-anchored spike protein guides coronavirus entry into host cells [2]. The spike is present in two very different forms: pre-fusion (the form on mature virions) and post-fusion (the form after membrane fusion has been completed). The pre-fusion structure is a homo-trimer, with three receptor-binding S1 heads sitting on top of a trimeric membrane-fusion S2 stalk [6–12]. The post-fusion structure is a coiled-coil structure, containing S2 only [13, 14]. The pre-fusion form is a metastable state: S2 is prevented from transitioning to the post-fusion structure due to the structural constraints imposed by S1. During cell entry, however, the spike is cleaved sequentially by host proteases at two sites: first at the S1/S2 boundary (i.e., S1/S2 site) and second within S2 (i.e., S2’ site) [15–17]. After the cleavages, S1 dissociates from S2, allowing S2 to transition to the post-fusion structure. The transition from pre-fusion to post-fusion form is irreversible, and hence this process is tightly regulated during the entry process [2].

Receptor binding is part of the regulation mechanisms for the structural transition of coronavirus spikes. Each S1 subunit of the spike contains an N-terminal domain (S1-NTD) and a C-terminal domain (S1-CTD) [1]. Depending on the virus, one or both of these S1 domains can function as the receptor-binding domain (RBD). S1-CTD is located on the tip of the spike trimer and is known to recognize protein receptors [1]. For coronaviruses whose S1-CTD functions as the RBD, such as SARS coronavirus (SARS-CoV) and MERS coronavirus (MERS-CoV), their S1-CTD constantly transitions between two conformations: standing up and lying down. Receptor binding stabilizes the S1-CTD in the standing-up conformation, weakening the S1/S2 interactions and facilitating the dissociation of S1 from S2 [8, 12, 18]. Thus, S1-CTD plays a double role in coronavirus entry: it determines viral attachment and facilitates membrane fusion. On the other hand, S1-NTD is located on the side of the spike trimer and mainly recognizes sugar receptors. To date S1-NTD has not been observed to undergo any dynamic conformational changes. Therefore, it is a mystery how S1-NTD would play any role in activation of the membrane fusion process, other than its established role in viral attachment.

MHV from the β-genus is an extensively studied prototypic coronavirus. MHV is the only known coronavirus that uses the S1-NTD to recognize a protein receptor, CEACAM1a [1, 19, 20]. CEACAM1a is a cell adhesion protein. Due to alternative mRNA splicing, CEACAM1a contains either two (D1-D4) or four (D1-D2-D3-D4) Ig-like domains [21]. Previously, we determined the crystal structure of MHV S1-NTD complexed with CEACAM1a (D1-D4) [22]. The structure showed that MHV S1-NTD has the same fold as human galectins (galactose-binding lectin), but it does not bind any sugar; instead, it binds to D1 of CEACAM1a through protein-protein interactions. The cryo-EM structures of MHV spike in pre-fusion and post-fusion have been determined [6, 13]. However, the structure of MHV spike in complex with CEACAM1a is still not available. As a result, although previous biochemical studies have shown that CEACAM1a binding triggers the conformational changes of MHV spike [23, 24], the molecular mechanism for the role of CEACAM1a in the MHV-spike-mediated membrane fusion is unknown.

In this study, we determined the cryo-electron microscopic (cryo-EM) structure of pre-fusion MHV spike in complex with CEACAM1a (D1-D4), which reveals the structural change of MHV spike associated with receptor binding. Using proteolysis and negative-stain EM assays, we further investigated the impact of receptor binding on proteases sensitivity and the final structural transitions of MHV spike. Our results provide insight into the molecular mechanism for MHV entry and demonstrate the diversity of entry mechanisms for different coronaviruses.

## Results

### Overall structure of MHV spike complexed with CEACAM1a

**Fig 1:**
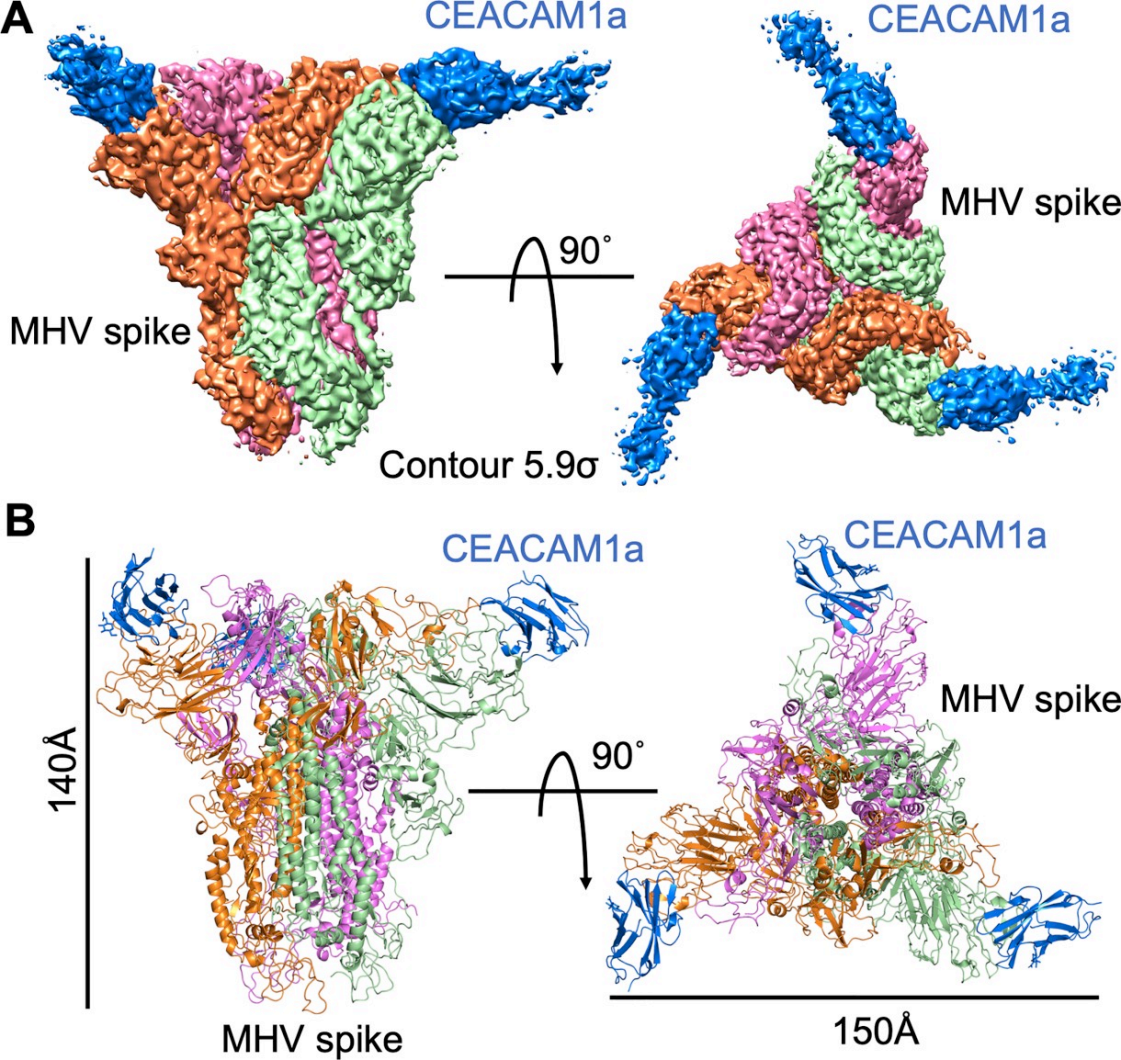
Overall structure of MHV spike protein/CEACAM1a complex. (A) Cryo-EM density map of MHV spike ectodomain/CEACAM1a complex. Left: side view. Right: top view. The trimeric MHV spike ectodomain (S-e) is in the pre-fusion state. Each monomeric subunit of MHV S-e is colored differently and CEACAM1A is colored in blue. (B) Atomic structure of MHV S-e/CEACAM1a complex. The molecules and subunits are colored in the same way as in panel (A). The views are also the same as in panel (A). The D4 domain of CEACAM1a had weak densities and hence its atomic model was not built.

We prepared both MHV spike ectodomain (S-e) and mouse CEACAM1a ectodomain (D1-D4) for cryo-EM studies. To prepare MHV S-e in the pre-fusion state, we removed the C-terminal transmembrane anchor and intracellular tail of MHV spike and replaced them with a GCN4 trimerization tag and a His_6_ tag. CEACAM1a was also engineered to contain a C-terminal His_6_ tag. Both MHV S-e and CEACAM1a were expressed in insect cells, secreted into cell culture medium, and purified to homogeneity using affinity column and size exclusion columns. Recombinant MHV S-e molecules were mostly intact and had not been cleaved by proteases. Subsequently recombinant MHV S-e and CEACAM1a were mixed together in solution and the complex was purified using a size exclusion column. We collected cryo-EM data on the complex and calculated a density map at 3.94 Å (Fig 1A, S1 Fig). The density of the complex revealed that each MHV S-e trimer binds three CEACAM1a molecules (Fig 1A). We built a structural model and refined it (Fig 1B). The final structural model contained all of the residues of the MHV S-e trimer (except residues 483–493, 832–853, and 1170–1227 in each monomer) as well as six N-linked glycans (two on each monomer). Although both the D1 and D4 domains of CEACAM1a could be seen in the cryo-EM density, only the density for the D1 domain was sufficiently robust for building the atomic model (Fig 1A, Fig 1B). Data collection and model statistics are shown in S1 Table.

**Fig 2:**
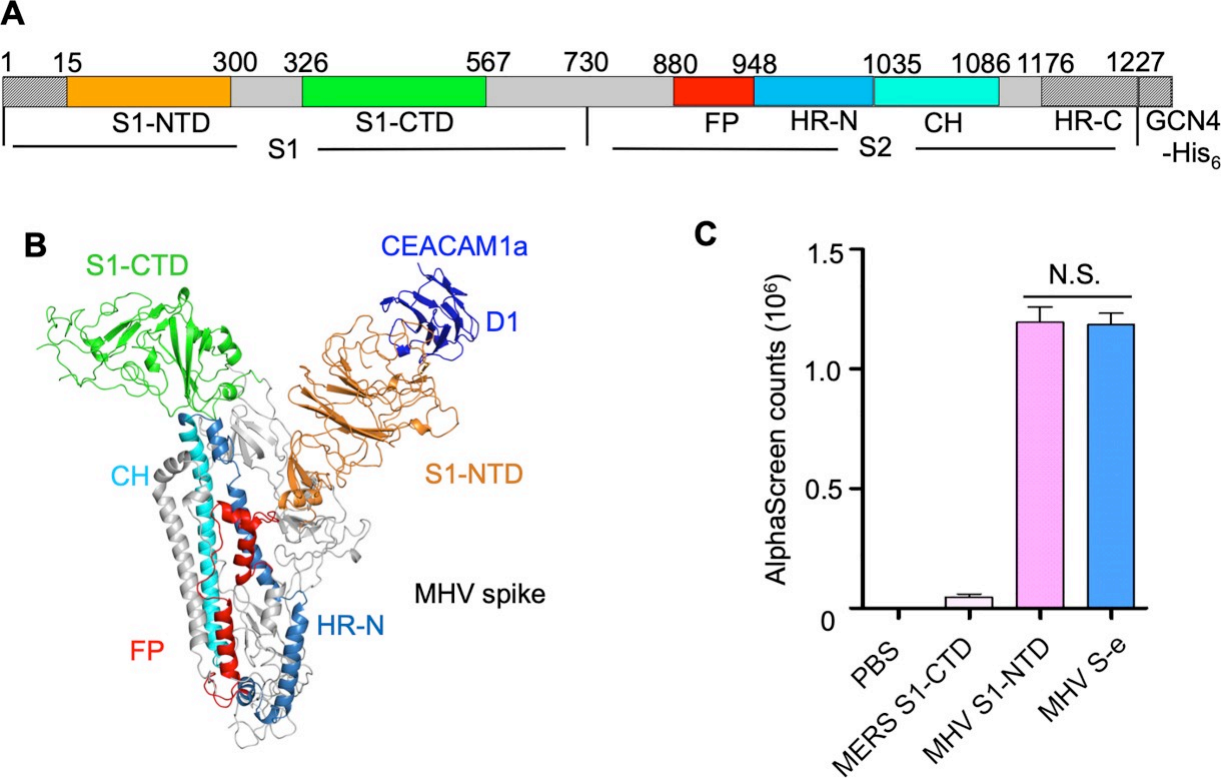
Detailed structure of MHV spike protein/CEACAM1a complex. (A) Schematic drawing of MHV S-e. S1: receptor-binding subunit. S2: membrane-fusion subunit. GCN4-His_6_: GCN4 trimerization tag followed by His_6_ tag. S1-NTD: N-terminal domain of S1. S1-CTD: C-terminal domain of S1. CH: central helix. FP: fusion peptide. HR-N and HR-C: heptad repeats N and C, respectively. (B) Structure of a monomeric subunit of MHV S-e/CEACAM1a complex. The structural elements of MHV S-e are colored in the same way as in panel (A). CEACAM1a is colored in blue. (C) Binding interactions between recombinant CEACAM1a (with a C-terminal Fc tag) and recombinant MHV S1-NTD or recombinant MHV S-e (with a C-terminal His_6_ tag) were measured using AlphaScreen assay. PBS and MERS-CoV S1-CTD, neither of which binds CEACAM1a, served as negative controls for MHV S-e and MHV S1-NTD. The error bars indicate standard deviation (SD) (n = 5). N.S.: statistically not significant (P > 0.05 in two tailed t-test).

The overall structure of the receptor-bound MHV S-e is similar to that of the unliganded S-e in the pre-fusion state. Like the unliganded S-e, the receptor-bound S-e contains three monomeric units, with three S1 heads sitting on top of the trimeric S2 stalk (Fig 2A, Fig 2B). Three copies of S1-CTD are located on the top of the trimer, all of which are in the lying down state. There are significant differences in the structural models of S1-CTD in the receptor-bound S-e and unliganded S-e; however, we believe that these differences are due to the improved cryo-EM density in the current study, which helped correct the misbuilt structural model in the unliganded S-e from an earlier study [6]. The revised atomic structures of S1-CTD and a second region of S1 were listed in S2 Fig. In both the receptor-bound and unliganded S-e trimer molecules, three copies of S1-NTD are located on each side of S1 (Fig 1B, Fig 2B). The structures of receptor-bound S1-NTD and unliganded S1-NTD are highly similar to each other (S3 Fig). Each S2 subunit contains a central helix (CH) (which mediates trimerization of the S2 stalk), a fusion peptide (FP) (which consists of three α-helices and several connecting loops), and a heptad repeat N region (HR-N) (which consist of three α-helices and several connecting loops). All of these structural elements in S2 are in the pre-fusion state and would need to undergo dramatic structural changes in order to transition to the post-fusion state. As in the unliganded S-e, the heptad repeat C region (HR-C) was not observed in the receptor-bound S-e structure probably due to its disorderness. It is worth noting that compared with the unliganded S-e, the proteolysis sites (at the S1/S2 region and S2’ site) do not become more exposed in the receptor-bound S-e (S4 Fig). Overall, receptor binding does not trigger dramatic structural changes in MHV S-e, which still stays in the pre-fusion conformation.

### Unique features of receptor binding by MHV spike

Receptor binding by MHV S-e reveals several unique features of a coronavirus spike using its S1-NTD as the RBD, as compared with SARS-CoV spike that uses its S1-CTD as the RBD [25]. First, almost all of the trimeric S-e molecules bind three CEACAM1a molecules each, while almost all of the SARS-CoV S-e molecules only bind one or two ACE2 molecules each [14, 26]. This is probably due to the fact that the three copies of MHV S1-NTD are located on different sides of the spike trimer, are far from each other, and hence the three bound receptor molecules do not have steric clashes. In contrast, the three copies of SARS-CoV S1-CTD are all located on the top of the spike trimer and are near each other, leading to steric clashes between bound ACE2 molecules. Depending on the number of receptor molecules on host cell membranes, the high stoichiometry of receptor binding by MHV spike potentially allows efficient viral attachment to target cells.

Second, in both the receptor-bound and unliganded MHV S-e molecules, all of the three copies of the S1-NTD are fully exposed and completely accessible for receptor binding (Fig 1B, Fig 2B). We compared the receptor-binding affinities of recombinant S1-NTD and recombinant S-e using AlphaScreen assay. The result showed that there is no significant difference in the CEACAM1a-binding affinities between recombinant S1-NTD and recombinant S-e (Fig 2C), consistent with our structural observation. Therefore, MHV S1-NTD is primed to recognize and engage the receptor. In contrast, the S1-CTD on SARS-CoV spike is not accessible in the lying down state and only becomes available to recognize ACE2 in the standing up state. This difference between the receptor-binding modes of MHV S1-NTD and SARS-CoV S1-CTD is probably attributed to the different locations and orientations of the two RBDs. In this case, S1-CTD is the most protruding region on the entire spike molecule (and also on the live virus particle) and is directly exposed to the host immune system. Thus, the lying down state of SARS-CoV S1-CTD is likely an immune evasion strategy for the virus, which would counter the neutralization by RBD-targeting antibodies. Compared with S1-CTD, S1-NTD is less exposed and hence is under reduced immune pressure. The readily accessible receptor-binding sites in MHV spike also potentially allow efficient viral attachment to target cells.

**Fig 3:**
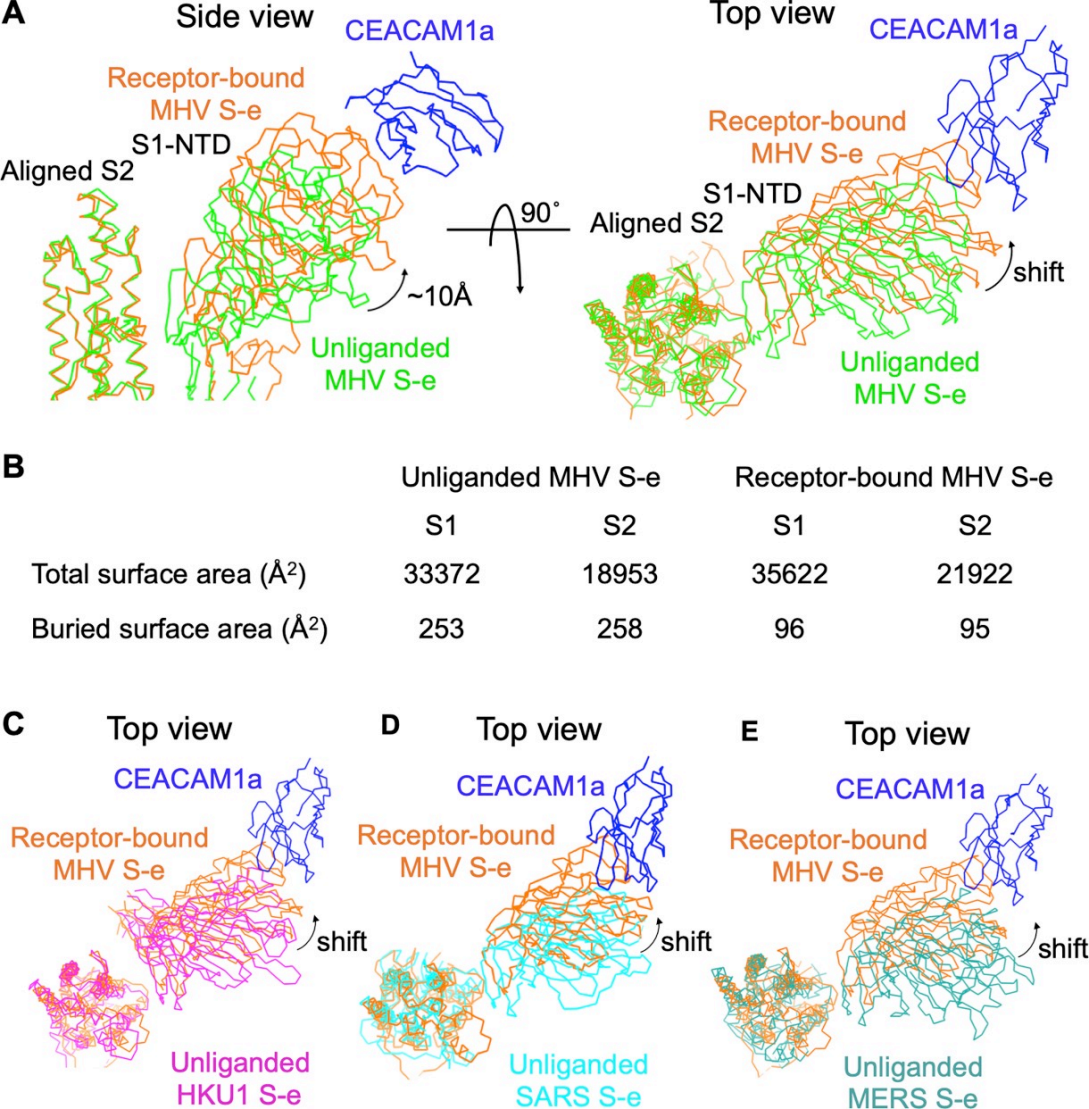
CEACAM1a-induced structural change of MHV spike. (A) Comparison of chain traces of S1-NTD in receptor-bound MHV S-e (colored in orange) and that in unliganded MHV S-e (colored in green), with the S2 subunits from the two S-e molecules aligned together. (B) Comparison of buried surface areas of S1 and S2 in receptor-bound MHV S-e trimer and unliganded MHV S-e trimer. Here the S1 and S2 are defined as regions before and after residue 730 (Fig 2A), respectively. (C-E) Same as in panel (A), except that unliganded MHV S-e is replaced by unliganded HKU1 S-e (PDB ID: 5I08; colored in magenta; panel C), unliganded SARS-CoV S-e (PDB ID: 5X5F; colored in cyan; panel D), or unliganded MERS-CoV S-e (PDB ID: 5X8; colored in dark green; panel E).

Third, CEACAM1a binding triggers structural changes in MHV S-e. Compared with the unliganded S-e, S1 in the receptor-bound MHV S-e moves up and away from the S2 subunit (Fig 3A). Specifically, there is ~10 Å movement of the edge of S1-NTD away from S2. Consequently, the interface between S1 and S2 in the receptor-bound S-e is significantly smaller than that in the unliganded S-e (Fig 3B). Specifically, before and after receptor binding, the buried interfaces of S1 and S2 decreased from 253 Å^2^ to 96 Å^2^ and from 258 Å^2^ to 95 Å^2^, respectively. Thus, CEACAM1a binding by MHV S1-NTD significantly reduces the interactions between S1 and S2. It is worth noting that despite containing misbuilt local regions in S1, the global structure of the unliganded MVH S-e was reliable [6]. Furthermore, we compared our structure of the receptor-bound MHV S-e with the unliganded S-e structures from other β-coronaviruses including HKU1, SARS-CoV, and MERS-CoV (Fig 3C, Fig 3D, Fig 3E). The results confirm our finding that compared with unliganded coronavirus S-e, the S1-NTD in the receptor-bound MHV S-e is farther away from the rest of the S1 structure, leading to more loosely packing of the spike trimer. As an interesting comparison, for SARS-CoV S-e, ACE2 binding also reduces the interactions between S1 and S2, but this is achieved through stabilization of the S1-CTD in the standing up position by ACE2 [8, 12]. Nevertheless, as in the case of SARS-CoV, the reduced interactions between S1 and S2 through receptor binding by MHV spike potentially facilitate the dissociation of S1 from S2 in the later membrane-fusion process (which we have verified below using biochemical studies).

Lastly, because S1-NTD is located on the side of spike trimer, the orientation of the spike-bound CEACAM1a is perpendicular to MHV spike (S5 Fig). It is worth noting that in the current cryo-EM study, recombinant ectodomain of CEACAM1a was used. *In vivo*, however, cell-surface-anchored CEACAM1a would not be able to approach MHV spike from the angle that is perpendicular to the spike. In other words, cell-surface-anchored CEACAM1a would need to bend in order to bind MHV spike. Indeed, previous studies have shown that CEACAM1a and other cell adhesion molecules have flexible domain hinges and are prone to molecular bending [27, 28]. In contrast, for SARS-CoV, the spike-bound ACE2 aligns with the spike *in vitro* [12, 26]; hence, cell-surface-anchored ACE2 can simply approach viral-envelope-anchored spike head-on *in vivo*. Although hypothetical, the bending of CEACAM1a molecule *in vivo* potentially produces tension in the spike-receptor complex, which may also facilitate the dissociation of S1 from S2 in the later membrane-fusion process.

In summary, the unique features of receptor binding by MHV spike include the following: all of the three CEACAM21a-binding sites in MHV spike are readily accessible for the receptor and are fully occupied by CEACAM1a; receptor binding induces structural changes in the spike that weaken the interactions between S1 and S2; the orientation of bound receptor, which is perpendicular the spike *in vitro*, indicates potential bending of the receptor molecule *in vivo*. These results guided us to further investigate the molecular mechanism of MHV-spike-mediated cell entry as follows.

### Role of receptor binding in the final conformational change of MHV spike

**Fig 4:**
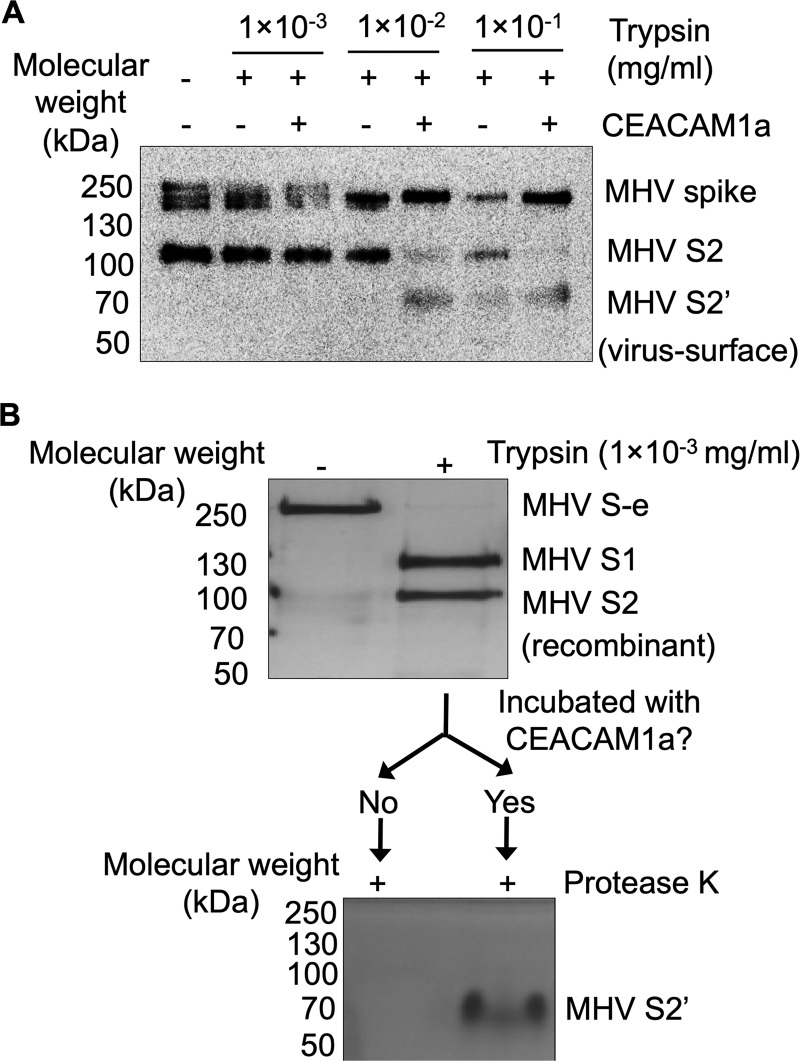
Receptor-facilitated proteolysis of MHV spike. (A) Western blot analysis of virus-surface MHV spike that had been cleaved by trypsin in the presence or absence of CEACAM1a. Different concentrations of trypsin were used. Here only protein fragments containing the C-terminal C9 tag (i.e., MHV spike, S2 and S2’, but not S1) could be detected since an antibody targeting the C-terminal C9 tag of MHV spike was used for the Western blot analysis. The result showed that receptor binding enhanced the protease sensitivity of MHV spike and produced more cleaved fragments (particularly S2’). (B) Silver staining analyses of recombinant MHV S-e that had been subjected to a double proteolysis assay. Specifically, recombinant MHV S-e molecules were first treated with low concentration of trypsin. Then half of the trypsin-cleaved products were incubated with CEACAM1a, while the other half were not. Subsequently both halves were treated with protease K. Here all protein fragments (i.e., MHV S-e, S1, S2 and S2’) could be detected as silver staining was used for the detection. The result showed that receptor treatment of the trypsin-cleaved MHV S-e led to a protease K-resistant S2’ fragment, suggesting that CEACAM1a binding facilitated the already cleaved MHV S-e to transition from pre-fusion to post-fusion conformation. See text for more discussion.

To examine the role of receptor binding in protease sensitivity of MHV spike, we performed proteolysis analysis of MHV spike in the presence or absence of CEACAM1a (Fig 4A). We packaged MHV spike into retrovirus particles (which lack their own envelope protein), producing MHV pseudoviruses. Subsequently, these MHV pseudovirus particles were incubated with different concentrations of trypsin in the presence or absence of CEACAM1a. Then the proteolysis fragments of MHV spike were examined using Western blot. The result showed that even without trypsin treatment, significant amounts of virus-surface MHV spike molecules had been cleaved to S2 fragment during the virus packaging process in human cells. This result is different from the uncleaved recombinant MHV S-e secreted from insect cells (Fig 4B), likely reflecting different protease activities in human and insect cells. At low trypsin concentrations, virus-surface MHV spike did not demonstrate additional proteolytic cleavage in the presence or absence of CEACAM1a (Fig 4A). At intermediate trypsin concentrations, virus-surface MHV spike was not further cleaved in the absence of CEACAM1a; however, a significant amount of virus-surface spike molecules were further cleaved to S2’ fragment in the presence of CEACAM1a (Fig 4A). At high trypsin concentrations, a small percentage of virus-surface spike molecules were further cleaved to S2’ fragment in the absence of CEACAM1a (Fig 4A). In contrast, a significant amount of virus-surface spike molecules were further cleaved to S2’ fragment in the presence of CEACAM1a (Fig 4A). As previous studies showed, the presence of the S2’ fragment is strongly associated with the final conformational change of coronavirus spikes [15, 23, 29]. Furthermore, it was previously demonstrated that MHV entry depends on the endosome pathway where lysosomal proteases play a critical role [30]. We recently showed that lysosomal extracts provide a good extracellular system for studying coronavirus entry through the endosome pathway [31]. Thus, to better mimic *in vivo* conditions, we repeated the above proteolysis assay, using cell-surface-expressed CEACAM1a (instead of recombinant CEACAM1a) and lysosomal extracts (instead of trypsin), and obtained similar results (S6 Fig). Therefore, although high concentrations of proteases inefficiently trigger the final conformational change of MHV spike, CEACAM1a binding significantly facilitates this process.

To further understand the role of receptor binding in protease sensitivity of MHV spike, we performed a two-step proteolysis experiment on MHV spike (Fig 4B). Specifically, recombinant MHV S-e was first cleaved into S1 and S2 fragments using trypsin. After stopping the trypsin reaction, the sample was split into two halves: one half was incubated with CEACAM1a, and the other was not. Then both halves were treated with protease K. The result showed that receptor treatment of the cleaved MHV S-e led to a protease K-resistant S2’ fragment. As shown below, MHV S-e that had been cleaved into S1 and S2 fragments remained in the pre-fusion conformation. Moreover, as discussed earlier, the protease K-resistant S2’ fragment likely represents the post-fusion conformation of coronavirus spikes [15, 23, 29]. Thus, CEACAM1a binding facilitated the already cleaved MHV S-e to transition from pre-fusion to post-fusion conformation, likely due to the removal of the structural restrain of S1 on S2 (in other words, dissociation of S1 from S2). We confirmed this result using virus-surface MHV spike (S7 Fig). These results are consistent with our structural observation showing that CEACAM1a binding to MHV spike weakens the interactions between S1 and S2.

### Negative-stain EM analysis of the final conformational change of MHV spike

**Fig 5:**
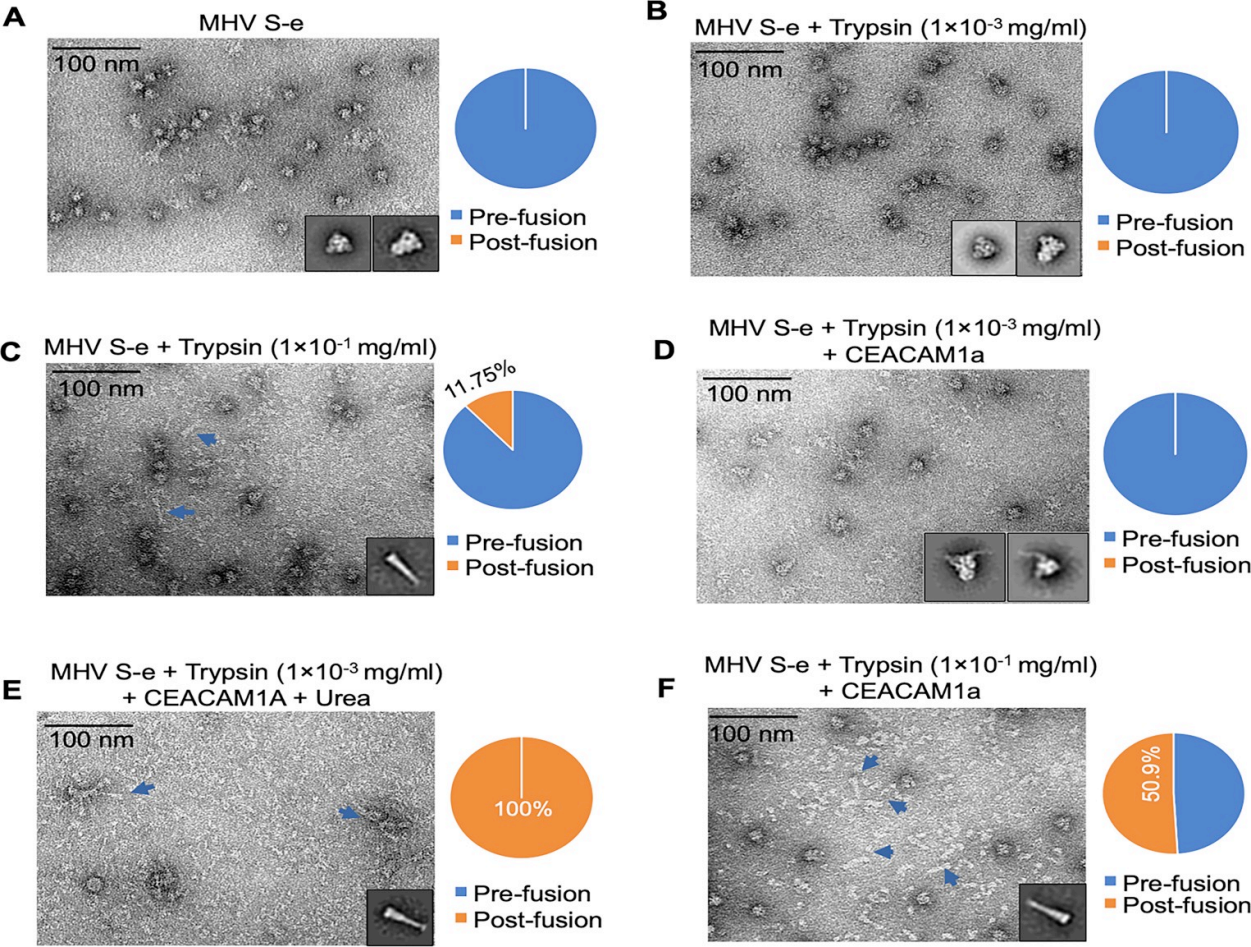
Negative-stain EM image of MHV spike treated with protease in the presence or absence of CEACAM1a. (A) MHV S-e without any protease treatment. All of the S-e molecules were in the pre-fusion state. (B) MHV S-e treated with low concentration of trypsin. All of the S-e molecules were in the pre-fusion state. (C) MHV S-e treated with high concentration of trypsin. 11.75% of the S-e molecules were in the post-fusion conformation (featured by the rod-like structure). (D) MHV S-e treated with low concentration of trypsin and incubated with CECAAM1a. All of the S-e molecules were in the pre-fusion state. (E) MHV S-e treated with low concentration of trypsin and incubated with urea. All of the S-e molecules were in the post-fusion state. (F) MHV S-e treated with high concentration of trypsin and incubated with CEACAM1a. 50.9% of the S-e molecules were in the post-fusion conformation. 2D averages of the S-e particles were shown as insets of each panel.

To directly view the final conformational change of MHV spike, we collected negative-stain EM images of recombinant MHV S-e that had been treated with trypsin. The results showed that without any treatment, MHV S-e stayed in the pre-fusion conformation (Fig 5A), which is consistent with our cryo-EM structure. Low concentration of trypsin did not trigger the final conformational change of MHV S-e (Fig 5B). However, high concentration of trypsin triggered 11.75% of the MHV S-e molecules to transition to the post-fusion conformation (Fig 5C). As previous studies showed, coronavirus spikes in the post-fusion conformation are rod-like structures containing S2 only; these rod-like structures represent the coiled-coil structures formed by the two heptad-repeat regions (i.e., HR-N and HR-C) of S2 [13, 14]. Moreover, because the hydrophobic fusion peptides become exposed in the post-fusion conformation, the post-fusion structures of coronavirus S2 tend to associate with each other on one end to form rosette-like structures. These negative-stain EM results are consistent with the proteolysis sensitivity results, both showing that high concentration of trypsin, but not low concentration of trypsin, can cleave a small percentage of MHV spike molecules to S2' fragments and trigger them to transition to the post-fusion conformation.

To investigate the role of CEACAM1a in the final conformational change of MHV spike, we collected negative-stain EM images of recombinant MHV S-e in the presence of recombinant CEACAM1a. The result showed that after being treated with low concentration of trypsin, all of the receptor-bound MHV S-e molecules remained in the pre-fusion conformation (Fig 5D). However, also after being treated with low concentration of trypsin, all of these receptor-bound MHV S-e molecules were triggered by urea to transition to the post-fusion conformation (Fig 5E). As shown by previous studies, urea (which is a denaturant) can facilitate the dissociation of coronavirus S1 from S2, leading to the final conformational change of coronavirus S2 [14]. Finally, after being treated with high concentration of trypsin, 50.9% of the receptor-bound MHV S-e molecules underwent the final conformational change and transitioned to the post-fusion conformation (Fig 5F). These negative-stain EM results are also consistent with the proteolysis sensitivity results, showing that CEACAM1a facilitates protease-cleaved MHV spike to undergo the final conformational change.

### Role of receptor binding in MHV cell entry

To analyze the role of CEACAM1a binding in MHV entry into host cells, we performed both MHV pseudovirus entry assay and live MHV infection assay (S8 Fig, S9 Fig). In both of these assays, virus particles were pretreated with both recombinant CEACAM1a and trypsin, and then subjected to entry into CEACAM1a-expressing cells. As a comparison, virus particles were pretreated with either recombinant CEACAM1a or trypsin before cell entry. The results showed that for both MHV pseudoviruses and live MHV, pretreatment with either recombinant CEACAM1a or trypsin reduced MHV entry into CEACAM1a cells. However, pretreatment with both recombinant CEACAM1a and trypsin further reduced MHV pseudovirus entry and even inactivated live MHV infection. As control experiments, MHV pseudoviruses did not enter cells not expressing CEACAM1a (except for the trypsin only condition where viral entry slightly increased). These results suggest that recombinant CEACAM1a alone could competitively inhibit MHV entry into CEACAM1a-expressing cells, trypsin alone could partially inactivate MHV spikes, and CEACAM1a and trypsin together drastically inactivate MHV spikes. Therefore, in addition to biochemical data, MHV cell entry data are also consistent with our structural observation showing that CEACAM1a binding to MHV spike weakens the interactions between S1 and S2 and facilitates the spike to be proteolysed.

## Discussion

Recent studies on coronavirus entry have been focused on those coronaviruses that use their S1-CTD as the receptor-binding domain. These studies have shown that S1-CTDs in those coronaviruses undergo a dynamic conformational change: lying down to evade immune surveillance and standing up for receptor binding [8, 12]. Receptor binding stabilizes S1-CTD in the standing up position, reducing the interface between S1 and S2. The weakened interactions between S1 and S2, plus two sequential protease cleavages (one at the S1/S2 boundary and the other at the S2’ site), allow S1 to dissociate from S2. Subsequently S2 undergoes the final conformational change and transitions to the post-fusion conformation. MHV differs from the above coronaviruses because it is the only coronavirus that uses its spike S1-NTD to bind a protein receptor. As a result of its unique receptor recognition pattern, the molecular mechanism for MHV entry is still elusive. In this study, we investigated the role of receptor binding by S1-NTD in the conformational changes of MHV spike, providing insight into the molecular mechanism for MHV entry.

**Fig 6:**
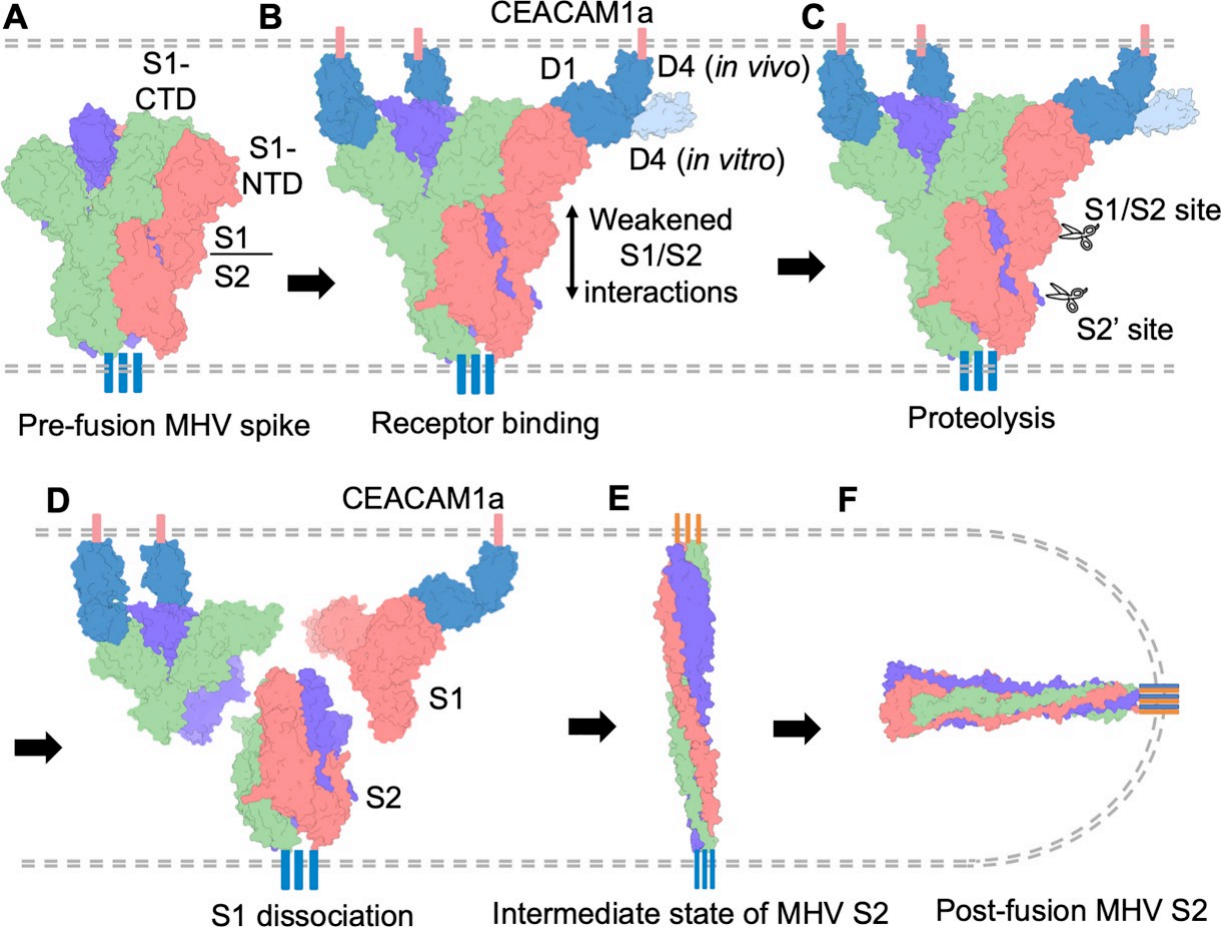
Proposed molecular mechanism of MHV entry. (A) Virus-surface MHV spike in the pre-fusion state. Each monomeric subunit of MHV spike trimer is colored differently. (B) Receptor binding by MHV spike. Host cell-surface CEACAM1a is colored in blue. Receptor binding triggers conformational changes in MHV spike, weakening the S1/S2 interactions. Although *in vitro* the receptor binds to MHV spike in an angle perpendicular to the spike, *in vivo* the receptor would need to bend in order to approach the receptor-binding sites in MHV spike. (C) Receptor-bound MHV spike is cleaved by proteases at two sites: S1/S2 site and S2' site. (D) Receptor facilitates S1 to dissociate from S2 through receptor-induced conformational changes in the spike, tension generated by potential bending of the receptor, and receptor-facilitated proteolysis of the spike. (E) Hypothetical intermediate state of MHV spike as proposed by many previous studies. (F) MHV spike transitions to the post-fusion state, leading to membrane fusion.

We performed a combination of structural and biochemical studies on the receptor-associated activities of MHV spike. These studies included determination of cryo-EM structure of receptor-bound MHV spike ectodomain, receptor-dependent protease sensitivity analysis of virus-surface MHV spike, negative-stain EM analysis of the receptor-facilitated conformational changes of MHV spike, and receptor-facilitated MHV cell entry. Based on our results, we propose the following molecular mechanism for MHV entry (Fig 6). During MHV entry into host cells, MHV spike binds to CEACAM1a on host cell surface for viral attachment. One spike is capable of binding three CEACAM1a molecules. Receptor binding triggers conformational changes in MHV spike, weakening the S1/S2 interactions and positioning MHV spike for two sequential proteolyses (one at the S1/S2 boundary and the other at the S2’ site). CEACAM1a, which has flexible domain hinges [27, 28], bends in order to approach S1-NTD on the side of the spike trimer. The receptor-induced conformational changes, receptor-facilitated proteolysis, and the potential bending of the receptor all contribute to the dissociation of S1 from S2. After S1 dissociates, S2 transitions to the post-fusion conformation through a hypothetical elongated intermediate state [32, 33].

The molecular mechanism for virus entry is one of the most fundamental questions in virology. Our study reveals the unique features of MHV entry, highlighting how receptor binding programs atomic level reorganization of MHV spike to promote membrane fusion. Hence MHV has adapted to its special need in receptor recognition and turns this need to its evolutionary advantage in cell entry. Our study demonstrates the diversity of cell entry by different coronaviruses and reveals new knowledge about this critical step in viral infection cycles.

## Materials and methods

### Expression and purification of MHV spike ectodomain and mouse CEACAM1a

MHV spike gene (strain A59) was kindly provided by Dr. Zhaohui Qian from Chinese Academy of Medical Sciences and Peking Union Medical College, Beijing, China. MHV spike ectodomain (S-e) (residues 15–1227) was cloned into pFastBac vector (Life Technologies Inc.); the construct contained an N-terminal honeybee melittin signal peptide and C-terminal GCN4 and His_6_ tags. It was expressed in Sf9 insect cells using the Bac-to-Bac system (Life Technologies Inc.) and purified as previously described [25]. Briefly, the protein was harvested from cell culture medium, and purified sequentially on Ni-NTA column and Superdex 200 size exclusion column (GE Healthcare). Mouse CEACAM1a ectodomain (residues 1–202) was expressed and purified as previously described [22, 34]; the construct contained a C-terminal His_6_ tag. Purified MHV S-e and CEACAM1a were mixed and incubate at 4°C for 2 hours. The MHV S-e/CEACAM1a complex was purified on Superdex 200 size exclusion column (GE Healthcare).

### Cryo-electron microscopy

For sample preparation, aliquots of the MHV S-e/CEACAM1a complex (3 μl, 0.35 mg/ml, in buffer containing 2 mM Tris pH7.2 and 20 mM NaCl) were applied to glow-discharged CF-2/1-4C C-flat grids (Protochips). The grids were then plunge-frozen in liquid ethane using a Vitrobot system (FEI Company).

For data collection, images were recorded using a Gatan K2 Summit direct electron detector in super resolution mode, attached to a FEI Titan-Krios TEM. The automated software SerialEM [35] was used to collect 2,250 total movies at 22,500x magnification and at a defocus range between 1 and 3 μm. Each movie had a total accumulated exposure of 77 e/Å2 fractionated in 50 frames of 10-second exposure. Data collection statistics are summarized in S1 Table.

For data processing, whole frames in each movie were corrected for motion and dose compensation using MotionCor2 [36]. ~1,800 best images were manually selected. The final images were bin-averaged to reach a pixel size of 1.06 Å. The parameters of the microscope contrast transfer function were estimated for each micrograph using GCTF [37]. Particles were automatically picked and extracted using RELION [38] with a box size of 320 pixels. Initially, 842,337 particles were extracted and subjected to 2D alignment and clustering using RELION. The best classes were then selected for an additional 2D alignment. ~5,000 best particles were selected for creating the initial 3D model using RELION. 210,067 particles selected from 2D alignment were then subjected to 3D classification. The best class with 82,923 particles was subjected to 3D refinement to generate the final density map. The final density map was sharpened with modulation transfer function of K2 operated at 300keV using RELION. Reported resolutions were based on the gold standard Fourier shell correlation (FSC) = 0.143 criterion. Fourier shell correction curves were corrected for the effects of soft masking by high-resolution noise substitution [39]. Data processing was concluded in S1A Fig.

### Model building and refinement

The initial model of the MHV S-e/CEACAM1a complex was obtained by fitting the cryo-EM structure of unliganded MHV S-e (PDB ID: 3JCL) and the crystal structure of MHV S1-NTD/CEACAM1a complex (PDB ID: 3R4D) into our cryo-EM density map using UCSF Chimera [40] and Coot *[41]*. Manual model rebuilding was performed using Coot based on the well-defined continuous density of the main chain. Side chain assignments were guided through the densities of N-linked glycans and bulky amino acid residues. The structural model of MHV S-e/CEACAM1a complex was refined using Phenix [42] with geometry restrains and three-fold noncrystallographic symmetry constraints. Refinement and model rebuilding were carried out iteratively until no further improvements were achieved in geometry parameters and model-map correlation coefficient. The quality of the final model was analyzed with MolProbity [43] and EMRinger [44]. The validation statistics of the structural models are summarized in S1 Table.

### AlphaScreen protein-protein binding assay

AlphaScreen protein-protein binding assay was carried out between recombinant MHV S1-NTD and recombinant CEACAM1a and between recombinant MHV S-e and recombinant CEACAM1a as described previously [45, 46]. Briefly, Fc-tagged CEACAM1a (at 6 nM final concentration) was incubated with either His_6_-tagged MHV S1-NTD or His_6_-tagged MHV S-e (at 100 nM final concentration) in ½ AreaPlate (PerkinElmer, Waltham, MA) at room temperature for 1 hour. AlphaScreen Nickel Chelate Donor Beads and AlphaScreen Protein A Acceptor Beads (PerkinElmer) were then added to one of the mixtures at final concentrations of 5 μg/mL each. The mixtures were then incubated at room temperature for 1 hour away from light. The AlphaScreen signals were measured using an EnSpire plate reader (PerkinElmer).

### Packaging of MHV pseudoviruses

MHV pseudoviruses were packaged as previously described [31, 47]. Briefly, full-length MHV spike gene (which contained a C-terminal C9 tag) was inserted into pcDNA3.1 (+) plasmid. Retroviruses pseudotyped with MHV spike and expressing a luciferase reporter gene were prepared through co-transfecting HEK293T cells with a plasmid carrying Env-defective, luciferase-expressing HIV-1 genome (pNL4-3.luc.RE) and the plasmid encoding MHV spike. The produced MHV pseudoviruses were harvested 72 hours post transfection.

### MHV pseudovirus entry assay

MHV pseudoviruses (strain A59) were generated as described above. The produced pseudoviruses with indicated treatment were then used to enter HEK293T cells expressing CEACAM1a. After incubation at 37°C for 5 hours, medium was changed and cells were incubated for an additional 60 hours. Cells were then washed with PBS and lysed. Aliquots of cell lysates were transferred to Optiplate-96 (PerkinElmer), followed by addition of luciferase substrate. Relative light unites (RLUs) were measured using EnSpire plate reader (PerkinElmer). All the measurements were carried out in triplicates.

### Proteolysis assay

MHV pseudoviruses were purified using a 10–30% sucrose gradient ultracentrifugation at 250,000×g at 4°C for 2 hours. Purified MHV pseudoviruses were incubated alone or with recombinant CEACAM1a (which is in excess) at 37°C for 30 minutes. Then MHV pseudoviruses were incubated with different concentrations of trypsin at 4°C for 30 minutes. Subsequently soybean trypsin inhibitor (which is in excess) was added to stop the reaction. Samples were then applied for Western blot analysis using an antibody targeting the C-terminal C9 tag of MHV spike.

### Double proteolysis assay

Recombinant MHV S-e molecules (3 μg) were first treated with low concentration of trypsin at room temperature for 10 min. The reactions were stopped using soybean trypsin inhibitor. The products from this first proteolysis assay were analyzed using silver staining. They were then divided into two halves: one half was incubated with CEACAM1a at 37°C for 2 hours, and the other half was not incubated with CEACAM1a. Subsequently both halves were treated with proteinase K (final concentration for the assay: 1 μM) on ice for 20 min. The products from the second proteolysis assay were analyzed using silver staining.

Purified MHV pseudoviruses were also subjected to the same double proteolysis assay, except that Western blot (using an antibody targeting the C-terminal C9 tag of MHV spike) replaced silver staining in analyzing the proteolysis products from both proteolysis assays.

### Cleavage of MHV spike using lysosomal extracts

Lysosomal extracts from HEK293T cells were prepared according to the lysosome isolation kit procedure (Sigma-Aldrich) as previously described [31]. Briefly, HEK293T cells were harvested and washed with PBS buffer and then resuspended in 2.7 packed cell volumes (PCV) of extraction buffer. The cells were then broken in a 7-ml Dounce homogenizer using a loose pestle (i.e., pestle B) until 80% to 85% of the cells were broken (protease inhibitors from the kit were omitted in our procedure). The samples were centrifuged at 1,000 × g for 10 min, and the supernatants were transferred to a new tube and centrifuged at 20,000 × g for another 20 min. The supernatants were removed, and the pellets were resuspended in extraction buffer as the crude lysosomal fraction (CLF). The CLF was diluted in buffer containing 19% Optiprep density gradient medium solution and further purified using density gradient centrifugation at 150,000 × g for 4 hours to produce lysosomal extracts. For cleavage of MHV spike using lysosomal extracts, purified MHV pseudoviruses were incubated with membrane-bound CEACAM1a (i.e., HEK293T cells expressing CEACAM1a on the surface) for 1 hour and then were treated with lysosomal extracts at 37°C for 20 min. Subsequently, samples were denatured and analyzes using SDS-PAGE gel. Cleaved MHV spike molecules were detected by Western Blot using an anti-C9 tag antibody.

### Live MHV infection assay

MHV live virus particles (strain A59) were generated from an infectious clone, which is comprised of seven fragments maintained in pSMART (Lucigen) or pCR-XL-TopoA (Invitrogen) vectors and was amplified according to previously published protocols [48]. Viral stock was propagated in delayed brain tumor (DBT) cells and viral titers were determined using plaque titration. For live MHV infection, viruses with indicated treatment were used to infect DBT cells with a multiplicity of infection (MOI) of 0.05 PFU/cell for a one-hour adsorption period, followed by three washes with phosphate-buffered saline (PBS). Fresh medium was then added to each culture, and the infection was maintained at 37°C. Each condition was performed in triplicate. Microscope images were obtained 7 hours post infection.

### Negative-stain electron microscopy

The MHV S-e/CEACAM1a complex treated under different conditions was diluted to a final concentration of 0.02 mg/mL in 2 mM Tris-HCl pH7.2 and then loaded onto glow-discharged 400-mesh carbon grids (Electron Microscopy Sciences). Subsequently the grids were stained with 0.75% uranyl formate. All micrographs were collected at the University of Minnesota using a Tecnai G2 Spirit BioTWIN at 120 keV (FEI Company) and an Eagle 3.1 mega pixel CCD camera at 6,000 × nominal magnification. For 2D image averaging, particles were picked and extracted using RELION.

### Calculation of interface area

The total surface area and buried surface area of pre-fusion MHV S-e and MHV S-e/CEACAM1a complex were calculated using the PISA server at the European Bioinformatics Institute (http://www.ebi.ac.uk/pdbe/prot_int/pistart.html) [49]. For each trimeric S-e (unliganded or receptor-bound), a PDB file containing both S1 subunits and S2 subunits was submitted to the PISA server, and the total surface area and buried surface area on S1 and S2 were individually calculated.

### Data sharing

All data discussed in the paper will be made available to readers.

## Supporting information

S1 Table Data collection and model validation statistics. (DOCX)

S1 Fig Single particle reconstruction of MHV spike/CEACAM1a complex. (A) Brief procedure of the single particle reconstruction. The numbers of particles used for each step are in parentheses. (B) Gold-standard Fourier shell correlation (FSC) curves for the cryo-EM density of the complex. The resolution was set at 3.94 Å.
(TIF)

S2 Fig Corrected structural models in two regions of MHV spike. Listed are partial cryo-EM density maps with fitted model main chains in the current study (A and D) and previous study (B and E) [6]. Two regions are shown: S1-CTD (A and B) and another region in S1 (D and E). Also shown are the comparisons of the chain traces of the two models (C and F). In panels C and F, receptor-bound S-e is colored in orange and unliganded S-e is colored in cyan. Portions of density with details are shown for (A) and (D).
(TIF)

S3 Fig Comparison of MHV S1-NTD structures in different contexts. (TIF)

S4 Fig Comparison of the protease cleavage sites (both the S1/S2 site and S2’ site) among the receptor-bound MHV S-e, unliganded MHV S-e, and unliganded HKU1 S-e. The protease sites are colored in red. In the unliganded MHV S-e (PDB ID: 3JCL), the previously misbuilt S1/S2 site has been rebuilt based on the deposited cryo-EM density (see S2 Fig for more details). The S2’ site in the unliganded MHV S-e as well as the two protease cleavage sites in unliganded HKU1 S-e (PDB ID: 5I08) were not entirely built. Nevertheless, the result showed that the cleavages sites in all of these spike molecules are exposed.
(TIF)

S5 Fig Structure of spike-bound CEACAM1a. (A) Cryo-EM density map of MHV S-e/CEACAM1a complex (side view). The densities for both domains D1 and D4 of CEACAM1a can be seen, but the density for domain D4 is not good for model building. Hence only the atomic model of domain D1 was built. (B) Structural model of MHV S-e/CEACAM1a complex (side view). Here the structural model of both domains of CEACAM1a was “borrowed” from the crystal structure of MHV S1-NTD/CEACAM1a complex (PDB: 3R4D) and aligned to the current structure of MHV S-e/CEACAM1a complex. (C) Cryo-EM density map of MHV S-e/CEACAM1a complex (top view). (D) Structural model of MHV S-e/CEACAM1a complex (top view). In the current *in vitro* study, recombinant CEACAM1a binds to MHV spike in an angle perpendicular to the spike. However, in vivo, cell-anchored CEACAM1a would need to bend in order to approach MHV spike.
(TIF)

S6 Fig Cell-surface-anchored CEACAM1a facilitates proteolysis of MHV spike by lysosomal extracts. Cell-surface-expressed CEACAM1a and lysosomal extracts replace recombinant CEACAM1a and trypsin, respectively, in Fig 4A. Protein fragments containing the C-terminal C9 tag (i.e., MHV spike, S2 and S2’, but not S1) could be detected by an antibody targeting the C-terminal C9 tag of MHV spike. The result showed that membrane-bound receptor enhanced the sensitivity of MHV spike to lysosomal proteases, producing more S2’ fragments.
(TIF)

S7 Fig More evidence on receptor-facilitated proteolysis of MHV spike. The double proteolysis assay was performed in the same way as in Fig 4B, except that MHV pseudoviruses were used instead of recombinant MHV S-e. Accordingly, Western blot analysis of virus-surface MHV spike fragments instead of silver staining of recombinant MHV spike fragments was used for detection of the proteolysis products. As a result, only protein fragments containing the C-terminal C9 tag (i.e., MHV spike, S2 and S2’, but not S1) could be detected. The result is consistent with that from Fig 4B.
(TIF)

S8 Fig Role of receptor binding in MHV pseudovirus entry. MHV pseudoviruses were pretreated with (i) only trypsin at 37°C for 10 min (the reaction was stopped by trypsin soybean inhibitor), (ii) only CEACAM1a, or (iii) CEACAM1a at 37°C for 1 hour followed by trypsin treatment at 37°C for 10 min (the reaction was stopped by trypsin soybean inhibitor). Subsequently the above MHV pseudoviruses were used to enter CEACAM1a-expressing cells, and the entry efficiency was characterized through luciferase signals accompanying entry. Cells not expressing CEACAM1a were used as negative controls. The final concentrations of the proteins in the assay are indicated in the figure.
(TIF)

S9 Fig Role of receptor binding in live MHV entry. Live MHV viruses were pretreated in the same way as in S8 Fig. Subsequently the above MHV viruses were used to enter CEACAM1a-expressing cells. Cytopathic effect (CPE) microscope images of infected cells were taken 7 hours post infection. The final concentrations of the proteins in the assay were the same as in S8 Fig.
(TIF)
