# Supplementary material for: Structure of mouse coronavirus spike protein complexed with receptor reveals mechanism for viral entry
Source: PLoS Pathog. 2020 Mar 9;16(3):e1008392. doi: 10.1371/journal.ppat.1008392 (PMC7082060; doi:10.1371/journal.ppat.1008392)
Supplement: S1 Table — (DOCX) [file ppat.1008392.s001.docx]

**S1 Table. Data collection and model validation statistics**

| **Data Collection** |  |
| --- | --- |
| Microscope | Titan Krios |
| Voltage (kV) | 300 |
| Camera | Gatan K2 |
| Camera model | Super-resolution |
| Defocus range (µm) | 1~3 |
| Exposure time (s) | 10 |
| Movies | 2250 |
| Frames per movie | 50 |
| Dose rate (*e*^-^/Å^2^/s) | 1.54 |
| Magnified pixel size (Å) | 0.53 |
| **Reconstruction** |  |
| Software | RELION 2.0 |
| Symmetry | C3 |
| Particles refined | 82,923 |
| Map Resolution (Å) | 3.94 |
| Map sharpening *B*-factor (Å^2^) | -150 |
| **Model Validation** |  |
| UCSF Chimera CC | 0.8723 |
| EMRinger Score | 1.80 |
| MolProbity Score | 2.04 |
| All-atom clashscore | 9.44 |
| C_β_ deviations | 0 |
| Rotamer outliers (%) | 0.95 |
| Ramachandran |  |
| Favored (%) | 90.28 |
| Allowed (%) | 9.64 |
| Outliers (%) | 0.08 |
| RMS deviations |  |
| Bond length (Å) | 0.008 |
| Bond angles (°) | 1.023 |
