# Supplementary figures and images for: Structure of mouse coronavirus spike protein complexed with receptor reveals mechanism for viral entry
Source: PLoS Pathog. 2020 Mar 9;16(3):e1008392. doi: 10.1371/journal.ppat.1008392 (PMC7082060; doi:10.1371/journal.ppat.1008392)

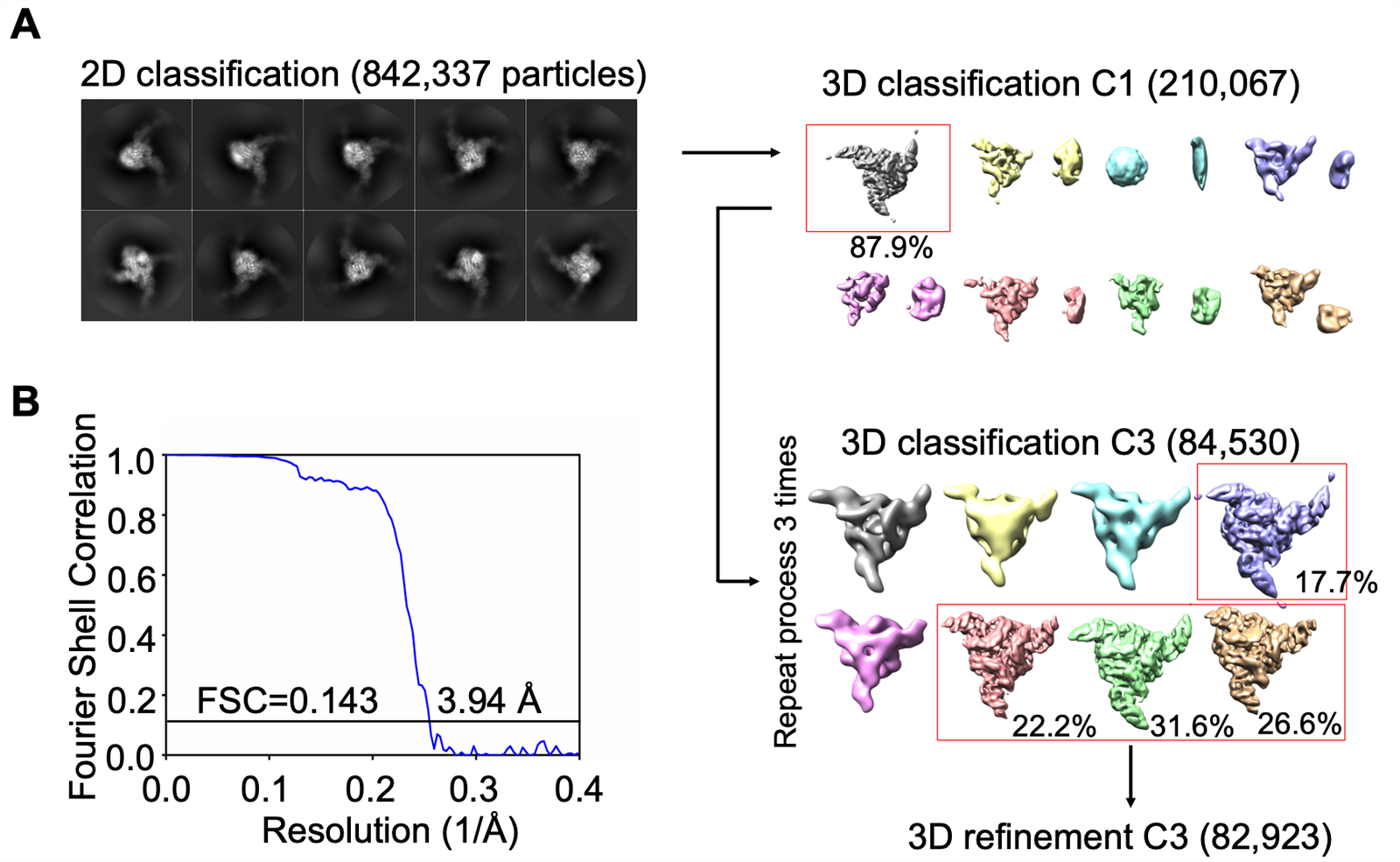

Supplement: S1 Fig — (A) Brief procedure of the single particle reconstruction. The numbers of particles used for each step are in parentheses. (B) Gold-standard Fourier shell correlation (FSC) curves for the cryo-EM density of the complex. The resolution was set at 3.94 Å. (TIF) [file ppat.1008392.s002.tif]

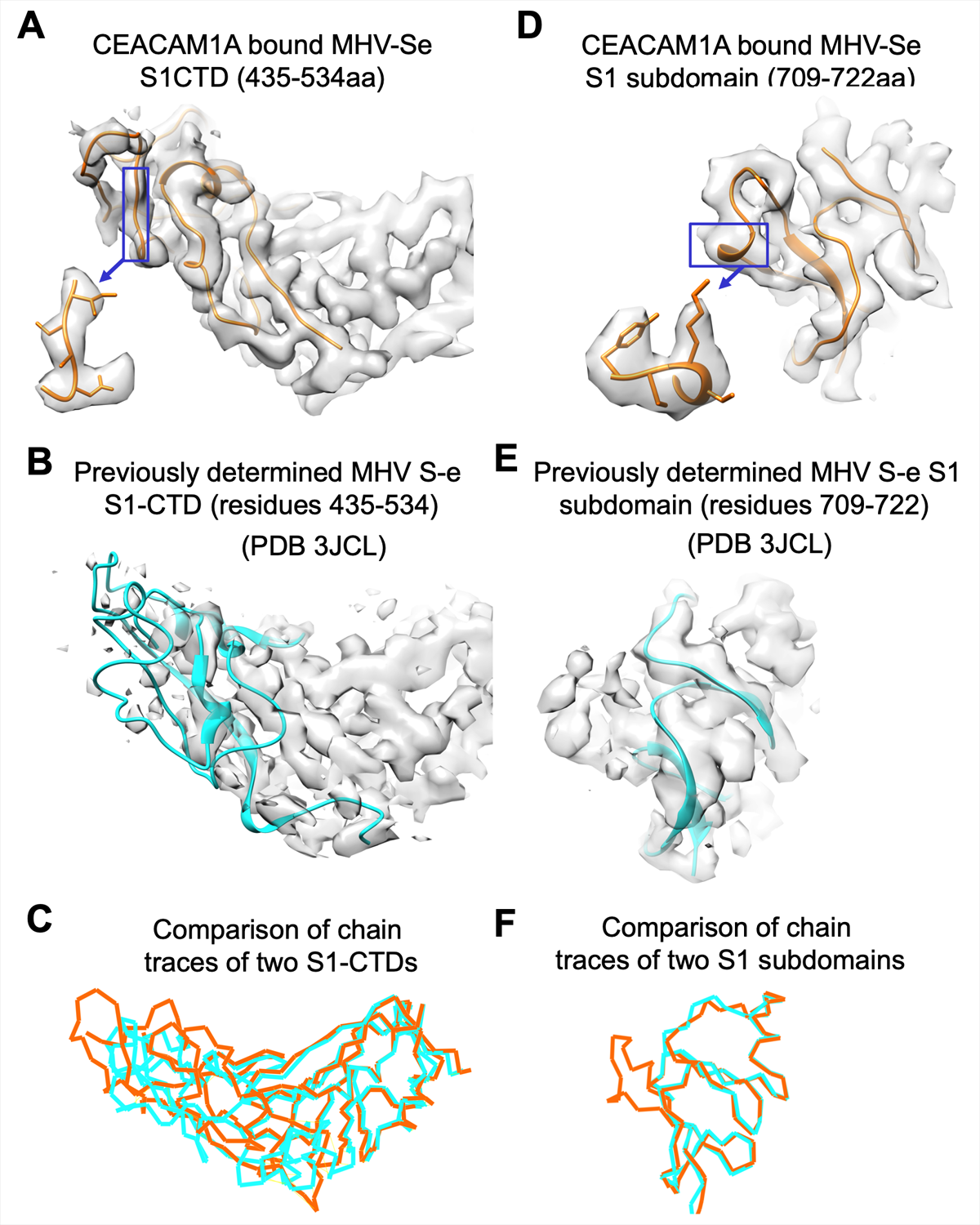

Supplement: S2 Fig — Listed are partial cryo-EM density maps with fitted model main chains in the current study (A and D) and previous study (B and E) [6]. Two regions are shown: S1-CTD (A and B) and another region in S1 (D and E). Also shown are the comparisons of the chain traces of the two models (C and F). In panels C and F, receptor-bound S-e is colored in orange and unliganded S-e is colored in cyan. Portions of density with details are shown for (A) and (D). (TIF) [file ppat.1008392.s003.tif]

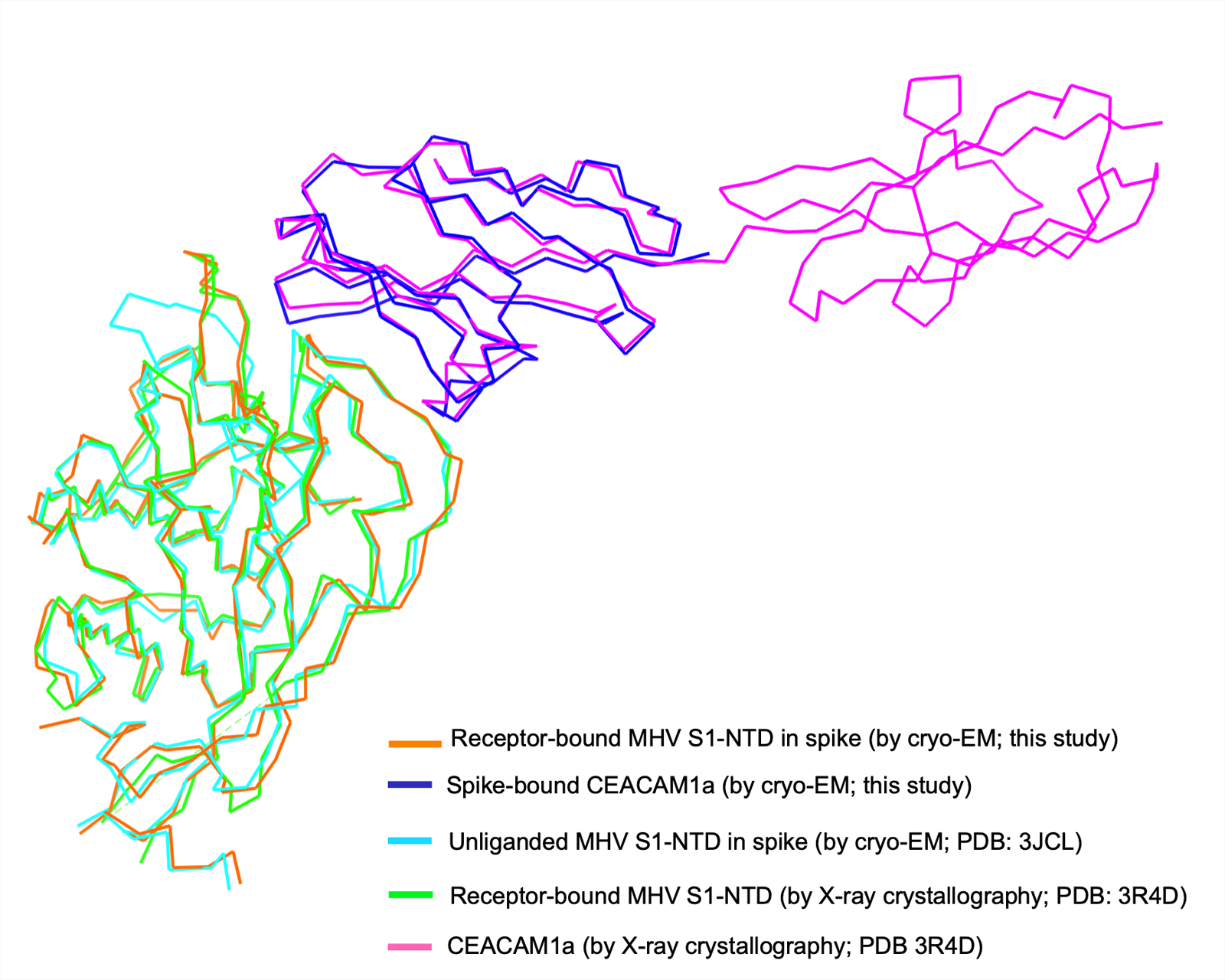

Supplement: S3 Fig — (TIF) [file ppat.1008392.s004.tif]

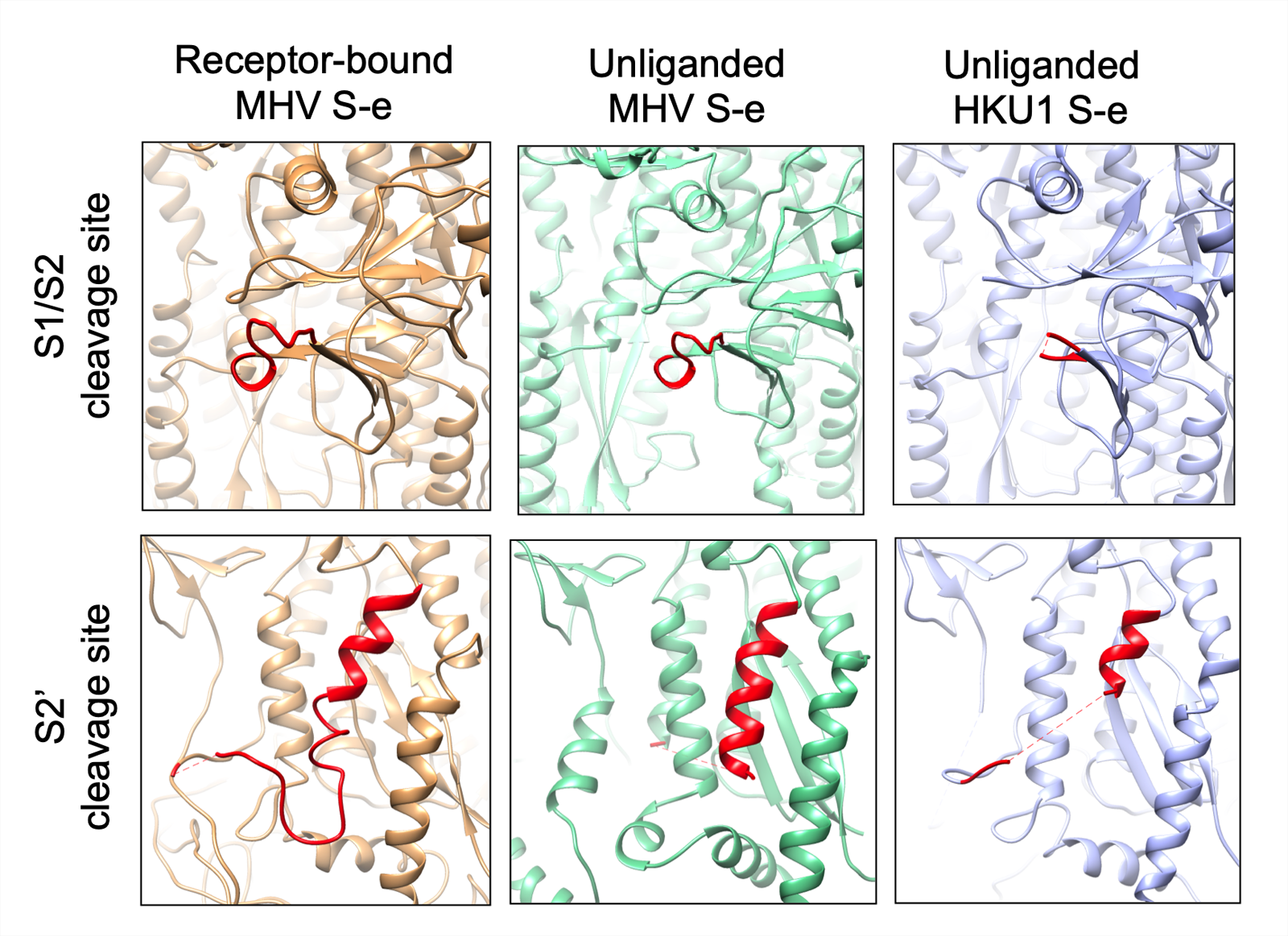

Supplement: S4 Fig — The protease sites are colored in red. In the unliganded MHV S-e (PDB ID: 3JCL), the previously misbuilt S1/S2 site has been rebuilt based on the deposited cryo-EM density (see S2 Fig for more details). The S2’ site in the unliganded MHV S-e as well as the two protease cleavage sites in unliganded HKU1 S-e (PDB ID: 5I08) were not entirely built. Nevertheless, the result showed that the cleavages sites in all of these spike molecules are exposed. (TIF) [file ppat.1008392.s005.tif]

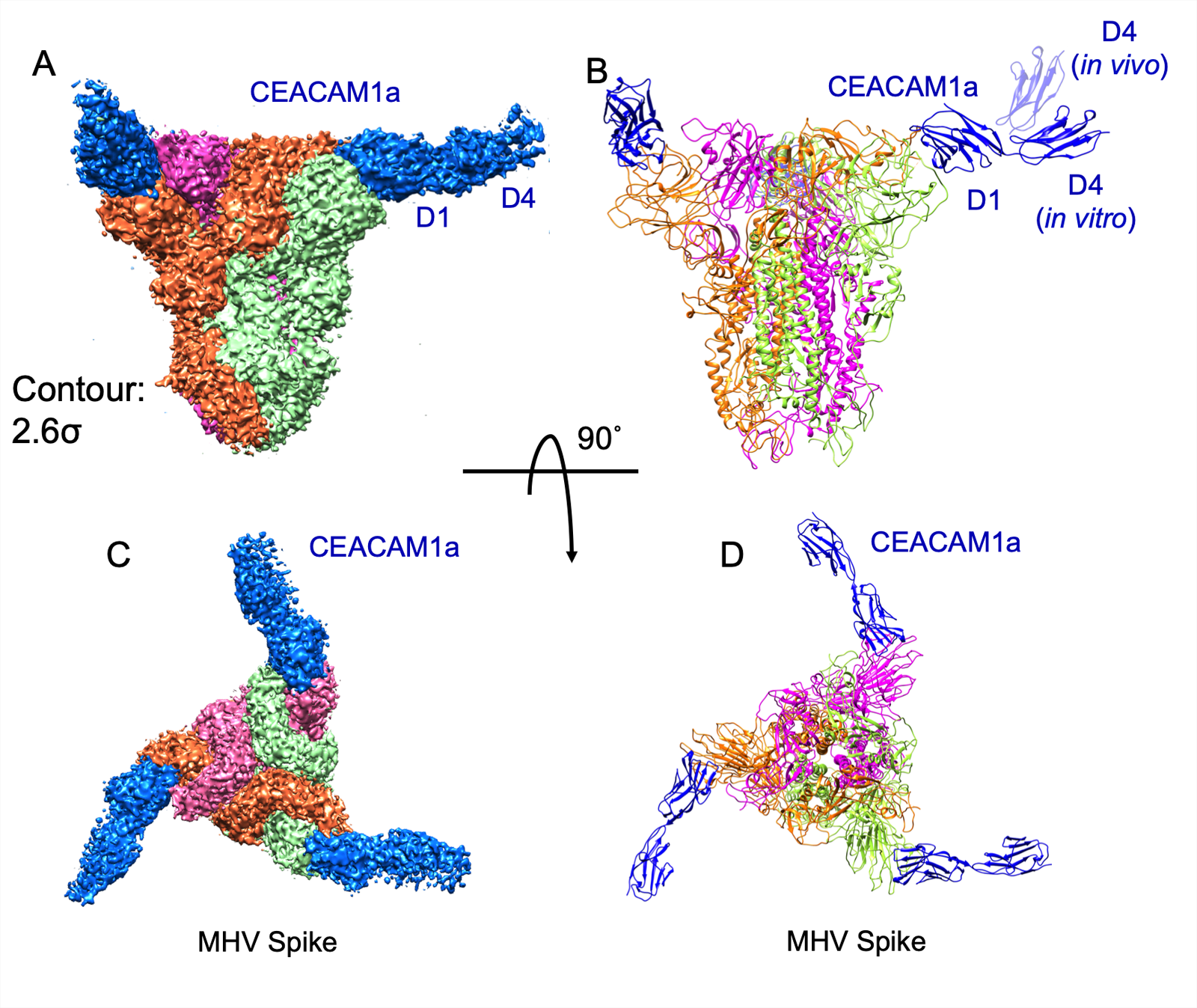

Supplement: S5 Fig — (A) Cryo-EM density map of MHV S-e/CEACAM1a complex (side view). The densities for both domains D1 and D4 of CEACAM1a can be seen, but the density for domain D4 is not good for model building. Hence only the atomic model of domain D1 was built. (B) Structural model of MHV S-e/CEACAM1a complex (side view). Here the structural model of both domains of CEACAM1a was “borrowed” from the crystal structure of MHV S1-NTD/CEACAM1a complex (PDB: 3R4D) and aligned to the current structure of MHV S-e/CEACAM1a complex. (C) Cryo-EM density map of MHV S-e/CEACAM1a complex (top view). (D) Structural model of MHV S-e/CEACAM1a complex (top view). In the current in vitro study, recombinant CEACAM1a binds to MHV spike in an angle perpendicular to the spike. However, in vivo, cell-anchored CEACAM1a would need to bend in order to approach MHV spike. (TIF) [file ppat.1008392.s006.tif]

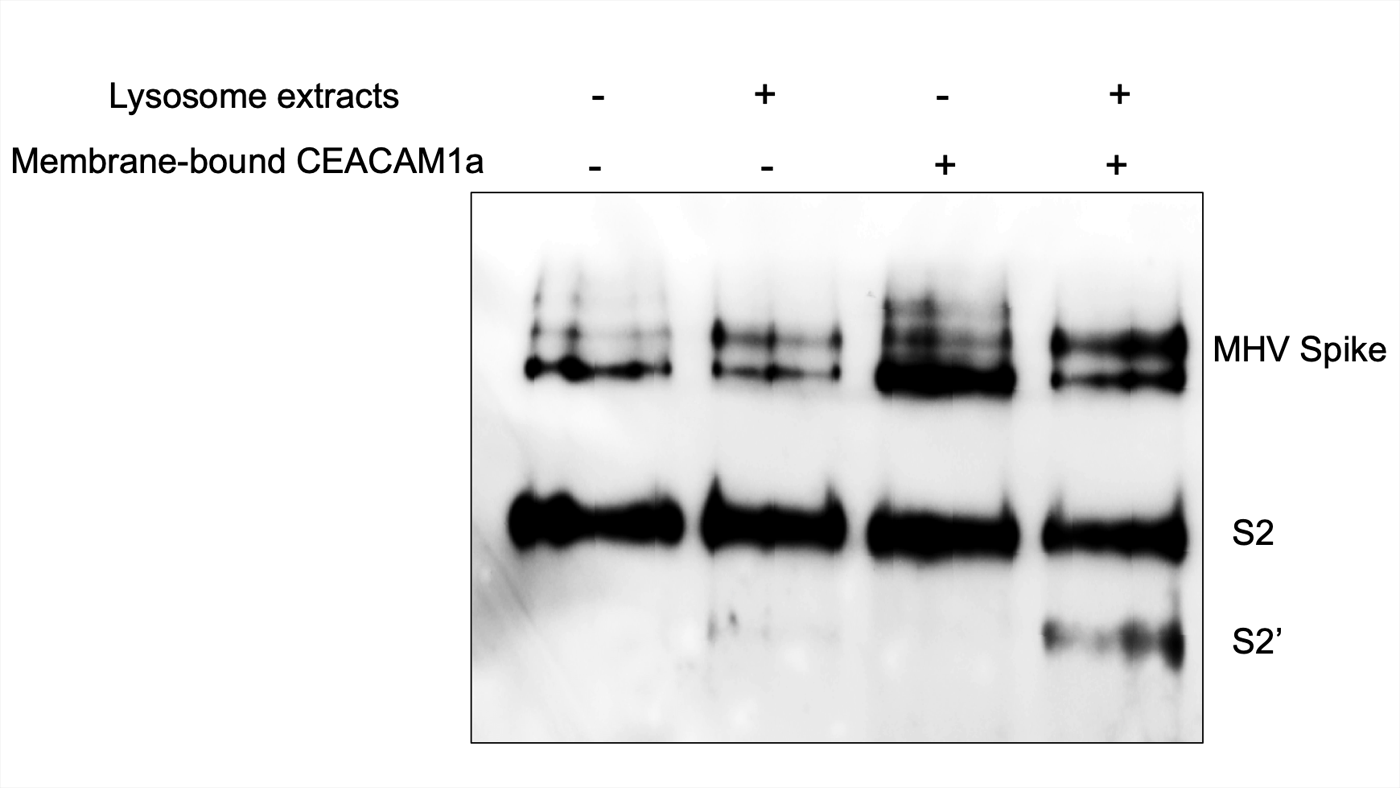

Supplement: S6 Fig — Cell-surface-expressed CEACAM1a and lysosomal extracts replace recombinant CEACAM1a and trypsin, respectively, in Fig 4A. Protein fragments containing the C-terminal C9 tag (i.e., MHV spike, S2 and S2’, but not S1) could be detected by an antibody targeting the C-terminal C9 tag of MHV spike. The result showed that membrane-bound receptor enhanced the sensitivity of MHV spike to lysosomal proteases, producing more S2’ fragments. (TIF) [file ppat.1008392.s007.tif]

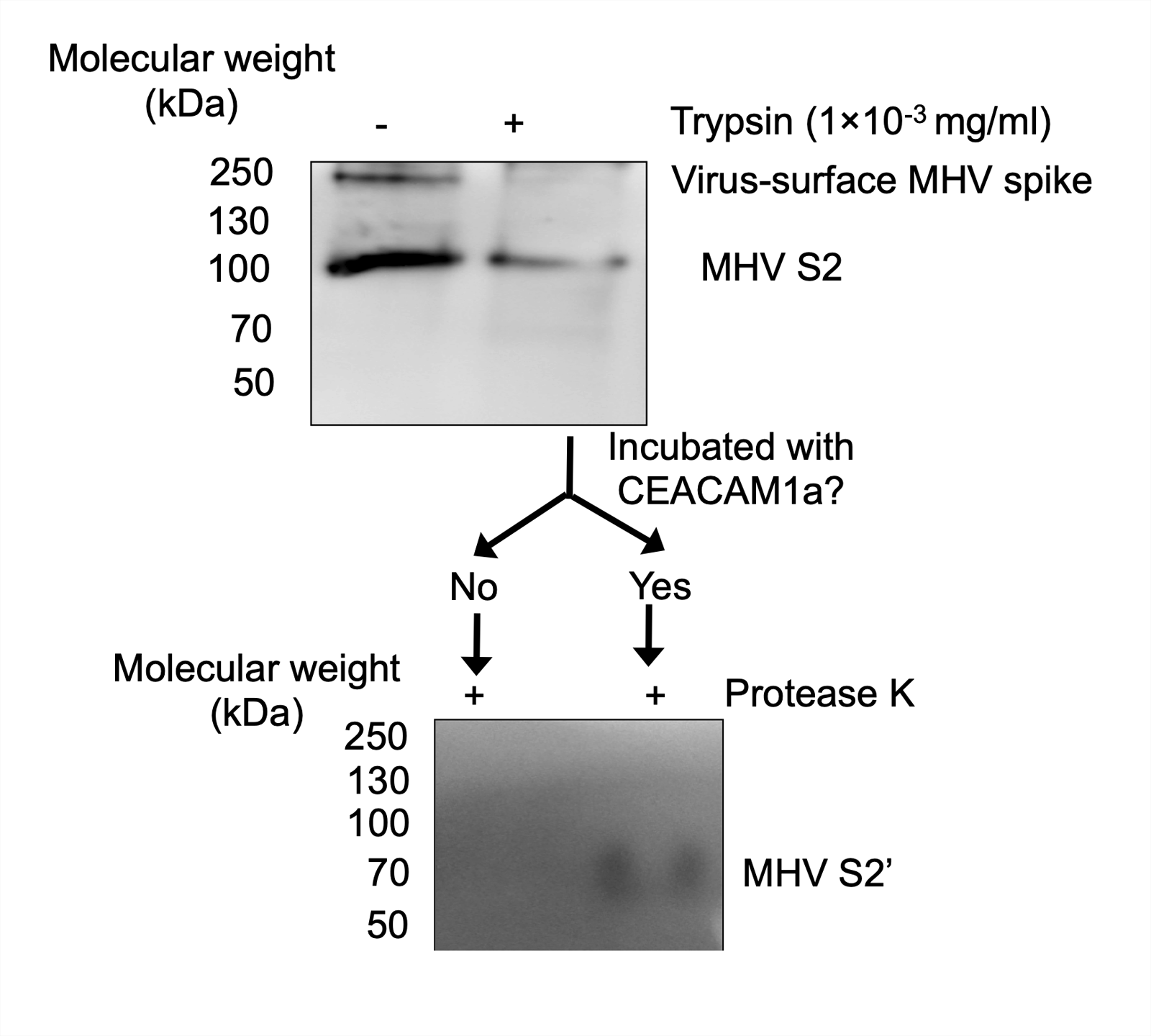

Supplement: S7 Fig — The double proteolysis assay was performed in the same way as in Fig 4B, except that MHV pseudoviruses were used instead of recombinant MHV S-e. Accordingly, Western blot analysis of virus-surface MHV spike fragments instead of silver staining of recombinant MHV spike fragments was used for detection of the proteolysis products. As a result, only protein fragments containing the C-terminal C9 tag (i.e., MHV spike, S2 and S2’, but not S1) could be detected. The result is consistent with that from Fig 4B. (TIF) [file ppat.1008392.s008.tif]

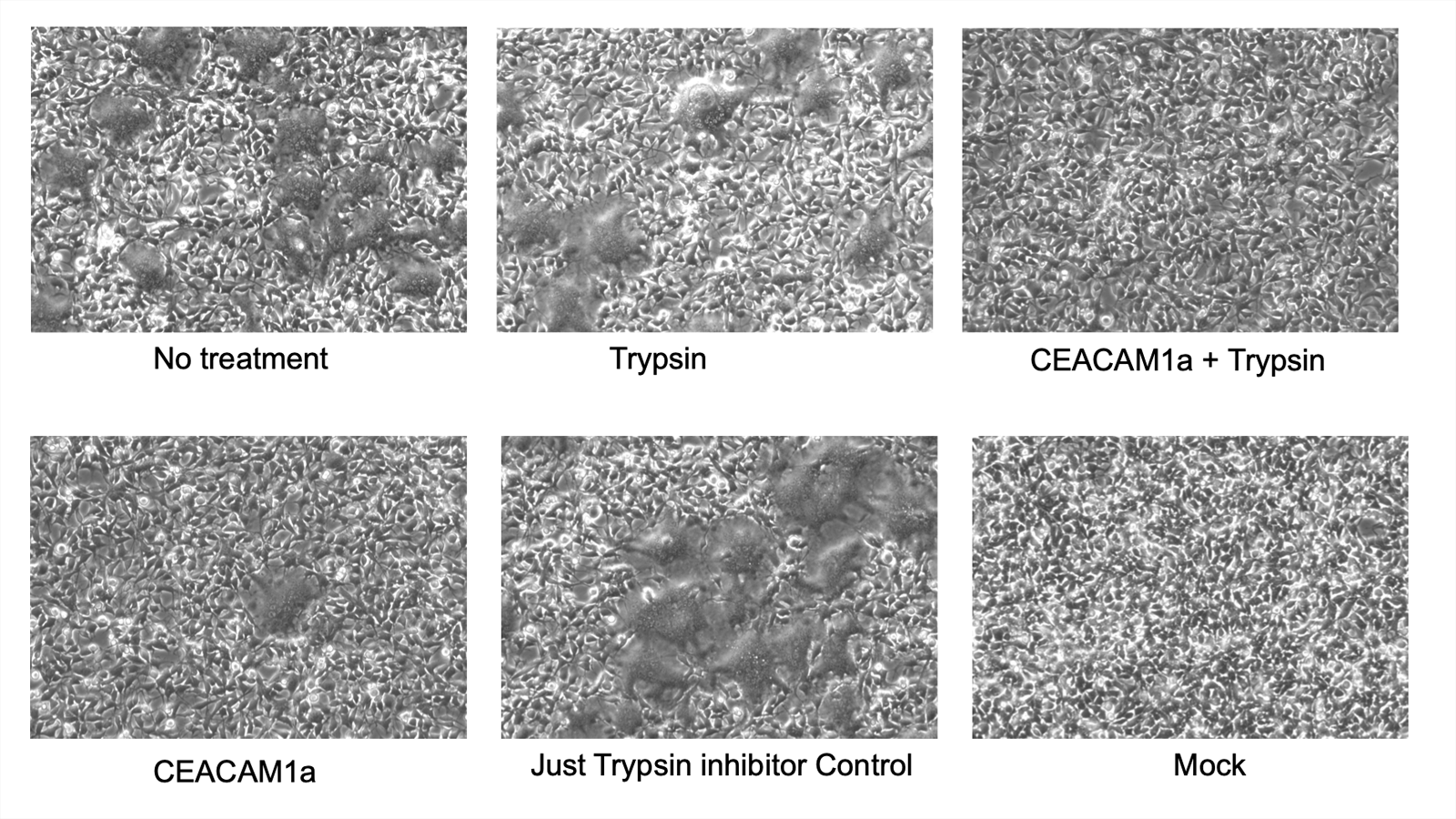

Supplement: S9 Fig — Live MHV viruses were pretreated in the same way as in S8 Fig. Subsequently the above MHV viruses were used to enter CEACAM1a-expressing cells. Cytopathic effect (CPE) microscope images of infected cells were taken 7 hours post infection. The final concentrations of the proteins in the assay were the same as in S8 Fig. (TIF) [file ppat.1008392.s010.tif]
